# Long non-coding RNAs regulate *Aedes aegypti* vector competence for Zika virus and reproduction

**DOI:** 10.1371/journal.ppat.1011440

**Published:** 2023-06-15

**Authors:** Alexandros Belavilas-Trovas, Spyros Tastsoglou, Shengzhang Dong, Mary Kefi, Mihra Tavadia, Kostas D. Mathiopoulos, George Dimopoulos

**Affiliations:** 1 W. Harry Feinstone Department of Molecular Microbiology and Immunology, Johns Hopkins Bloomberg School of Public Health, Baltimore, Maryland, United States of America; 2 Laboratory of Molecular Biology and Genomics, Department of Biochemistry & Biotechnology, University of Thessaly, Larissa, Greece; 3 DIANA-Lab, Department of Computer Science and Biomedical Informatics, University of Thessaly, Lamia, Greece; 4 Hellenic Pasteur Institute, Athens, Greece; Pennsylvania State University - Main Campus: The Pennsylvania State University - University Park Campus, UNITED STATES

## Abstract

Long non-coding RNAs (lncRNAs) play critical regulatory roles in various cellular and metabolic processes in mosquitoes and all other organisms studied thus far. In particular, their involvement in essential processes such as reproduction makes them potential targets for the development of novel pest control approaches. However, their function in mosquito biology remains largely unexplored. To elucidate the role of lncRNAs in mosquitoes’ reproduction and vector competence for arboviruses, we have implemented a computational and experimental pipeline to mine, screen, and characterize lncRNAs related to these two biological processes. Through analysis of publicly available Zika virus (ZIKV) infection-regulated *Aedes aegypti* transcriptomes, at least six lncRNAs were identified as being significantly upregulated in response to infection in various mosquito tissues. The roles of these ZIKV-regulated lncRNAs (designated *Zinc1*, *Zinc2*, *Zinc3*, *Zinc9*, *Zinc10* and *Zinc22*), were further investigated by dsRNA-mediated silencing studies. Our results show that silencing of *Zinc1*, *Zinc2*, and *Zinc22* renders mosquitoes significantly less permissive to ZIKV infection, while silencing of *Zinc22* also reduces fecundity, indicating a potential role for *Zinc22* in trade-offs between vector competence and reproduction. We also found that silencing of *Zinc9* significantly increases fecundity but has no effect on ZIKV infection, suggesting that *Zinc9* may be a negative regulator of oviposition. Our work demonstrates that some lncRNAs play host factor roles by facilitating viral infection in mosquitoes. We also show that lncRNAs can influence both mosquito reproduction and permissiveness to virus infection, two biological systems with important roles in mosquito vectorial capacity.

## Author summary

Long non-coding RNAs (lncRNAs) emerged during the last decades as important regulators of diverse biological processes. Despite their significance in various organisms, their role is still largely unexplored in mosquitoes that transmit human pathogens. Some of their functions in processes related to vectorial capacity could be exploited for the development of novel disease control strategies. Here we provide a comprehensive analysis of lncRNAs that regulate vector competence and reproduction of the notorious mosquito disease vector, *Ae*. *aegypti*. We show that the expression of several lncRNAs is stimulated by infection with Zika virus and that silencing three of them render mosquitoes more resistant to the virus infection, possibly through an immunity-related function. Moreover, we provide evidence regarding their impact on mosquitoes’ reproduction, such as fecundity and fertility. We also highlight the influence of *Zinc22*, an ovary-enriched lncRNA, that affects both reproduction and virus restriction, indicating its potential role in metabolic trade-offs between these two systems. This is the first study to analyze the influence of lncRNAs in vectorial capacity and reproduction of disease vectors, and thereby breaks new ground for their potential use in novel mosquito control and pathogen transmission-blocking strategies.

## Introduction

*Aedes aegypti* is a major mosquito vector of many arthropod-borne viruses (arboviruses), including dengue virus (DENV), Zika virus (ZIKV), and chikungunya virus (CHIKV). Arboviruses are mainly spread through a horizontal transmission cycle between an arthropod vector and a vertebrate host. Arboviruses that are transmitted by mosquito vectors enter the mosquito’s body after a viremic blood meal has been taken from a vertebrate host. First, they infect midgut epithelial cells, where they replicate and produce viral particles. They then escape the midgut basal lamina and disseminate to the hemocoel and finally infect salivary glands, from where they can be transmitted to another vertebrate host through saliva during the next blood meal [1]. The average extrinsic incubation period (EIP) of the major flaviviruses, such as DENV and ZIKV, ranges from 7 to 14 days.

Beyond the physical barriers that protect against pathogens, mosquitoes mainly rely on their innate immune system to defend against arboviral infections [2]. Anti-viral defenses can be regulated by at least five distinct signaling pathways: the RNA interference (RNAi), Toll, immune deficiency (IMD), Janus kinase-signal transducer and activator of transcription (JAK-STAT), and mitogen-activated protein kinase (MAPK) pathways [3–6]. RNAi is one of the best described antiviral mechanisms relying on Dcr2, R2D2 and Ago2, the components of the RNA-induced silencing complex (RISC) [7] that mediate cleavage of the viral RNA [8]. Some factors of the other antiviral pathways have also been characterized, such as DENV restriction factors 1 & 2 (DVRF1, DVRF2) of the JAK/STAT pathway [9] and Rel1 and MyD88 of the Toll pathway [5,10]. However, many aspects of insect immune response against arboviruses are still not well understood. Moreover, viruses rely on numerous mosquito-encoded host factors to establish their replication and infection. Even though several host factors, including a vATPase and IMPDH, have been described [4,11], this field is still largely unexplored.

Long non-coding RNAs (lncRNAs), non-coding transcripts with sequences longer than 200 nucleotides [12], have been associated with multiple cellular functions. These functions include, but are not limited to: (i) guiding transcription factors or other enzymes, (ii) decoying proteins, (iii) sponging up microRNAs (miRNAs), (iv) acting as precursors of miRNAs, and (v) functioning as scaffolds for ribonucleoprotein complexes (reviewed in [13]). There is also evidence that lncRNAs are involved in insect innate immunity. In *Drosophila melanogaster*, a lncRNA named viral suppressor of RNAi (VSR)-interacting RNA (VINR), regulates the innate immune response upon infection with the *Drosophila* C virus (DCV) [14]. The VSRs are virulence factors produced by viruses to evade RNAi defense mechanisms. In the absence of VINR, viral titers of DCV increase dramatically. Interestingly, once a dsRNA-binding VSR is detected, VINR activity leads to the upregulation of antimicrobial peptide (AMP) genes. *Induced by Infection* (IBIN) is a *D*. *melanogaster* lncRNA that has been found to be highly expressed after infection with *Micrococcus luteus*, a Gram-positive bacterium [15]. Its upregulation results in high expression of genes involved in the Toll pathway, such as the AMP Drosomycin and the Toll pathway target IM1. Its overexpression also results in the upregulation of *Npc2e*, a gene that is involved in antimicrobial responses, through the IMD signaling pathway [16].

While our understanding of lncRNA biology has advanced significantly through studies in *D*. *melanogaster*, their role in *Ae. aegypti* biology is still limited. A study that focused on the role of lncRNAs that are regulated in the *Ae. aegypti* Aag2 cells upon infection with dengue virus [17] showed that 421 lncRNAs were differentially expressed (DE), and RNAi-mediated silencing of lincRNA-1317 led to a slight enhancement of viral replication. Another study using the same cell line identified 3,035 differentially expressed lncRNAs in response to infection with *Wolbachia*. Silencing of two of them (*aae-lnc-7598* and *aae-lnc-0165*) revealed their influence on mosquito permissiveness to dengue virus. Specifically, *aae-lnc-7598* expression modulated the antioxidant catalase 1B (CAT1B), which enhances production of reactive oxygen species (ROS), and *aae-lnc-0165* activated REL1, a key regulator of the Toll pathway [18]. It is also likely that lncRNAs can influence infection by regulating the expression of virus host factors.

Beyond their role in immunity, lncRNAs have also been shown to regulate reproduction and may mediate trade-offs between reproduction and both vector competence and immunity. In many cases, increased reproductive effort results in reduced immunity and, reciprocally, infection and activation of the immune system reduce reproductive output. Several such findings have been reported in dipteran species, especially *D*. *melanogaster* [19–25] and the mosquitoes *Ae*. *aegypti* [26], *An*. *gambiae* [27,28] and *An*. *stephensi* [29]. Trade-offs are manifested by signaling mechanisms that are mainly regulated by a balance of the hormones 20-hydroxyecdysone (20E) and Juvenile hormone (JH) [30,31]. JH and 20E regulate the development of oogenesis and have been connected to ovarian follicle maturation and vitellogenesis [32]. JH and 20E also influence, in opposite ways, insect immunity. In *D*. *melanogaster*, JH has a negative effect, as it reduces the activation of AMPs [33], while 20E enhances the transcription of AMPs [33–36] and drives the expression of the PGRP-LC protein, which is an activator of the IMD immune pathway [37]. In *Ae*. *aegypti*, studies have revealed that JH and 20E modulate vitellogenesis and have a negative impact on both the expression of AMPs [38] and stimulation of the IMD pathway [39], respectively.

It is possible that lncRNAs involved in regulating vector competence of mosquitoes to arbovirus infection could be used to develop transmission-blocking strategies. Available transcriptomic data indicate that several lncRNAs are differentially expressed after infection with various arboviruses. However, published work that has aimed to characterize the role of specific lncRNAs in modulating virus infection has focused exclusively on the effect of infection in cell lines, and published *in vivo* data have been lacking regarding the influence of lncRNAs on the circulation and the infectivity of the virus in mosquitoes. Thus, the present study has been designed to identify specific lncRNAs that can regulate mosquito permissiveness to virus infection, possibly through antiviral immunity or other mechanisms, and assess the influence of such lncRNAs on female reproduction. These lncRNAs are likely to play roles in reproduction-vector competence and immunity trade-offs, and thereby represent potential disease control targets.

## Results

### Classification of lncRNAs in *Ae*. *aegypti*

**Fig 1:**
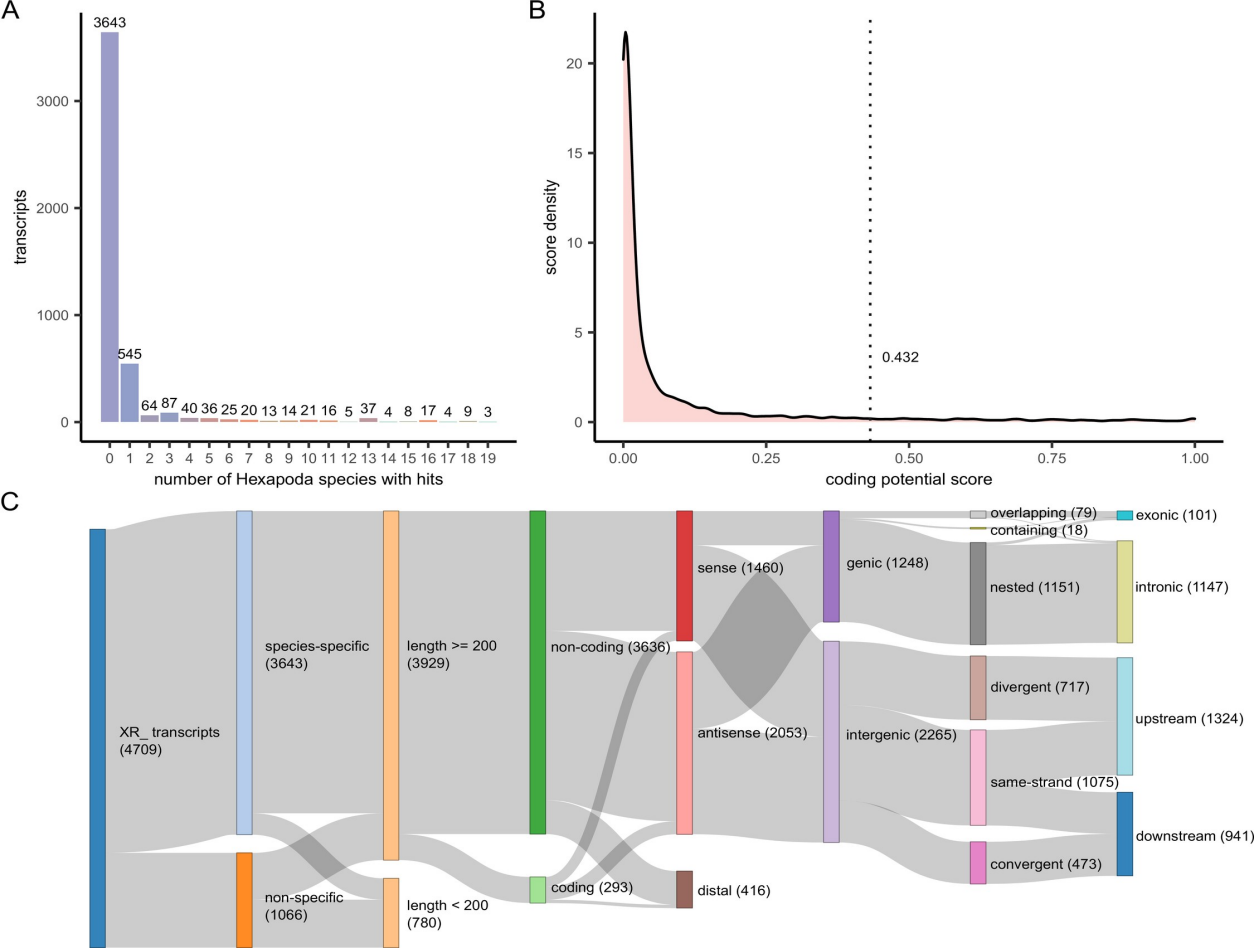
Conservation, coding probability, and genomic localization information for NCBI automatically annotated lncRNAs in *Aedes aegypti*. **(A)** The number of lncRNA sequences vs. the number of species against which they were found to exhibit Blast hits (top 20 hits shown). **(B)** Density plot of the coding potential model scores for all lncRNAs. The vertical dotted line indicates the cutoff point (0.432), below which transcripts were classified as non-coding. **(C)** Sankey plot displaying the overall annotation process and metrics for all RefSeq ncRNA transcripts (denoted by the “XR” prefix).

To identify *Ae*. *aegypti*-specific lncRNAs, we compared automatically annotated ncRNA transcript sequences of *Ae*. *aegypti* against all Hexapoda RefSeq RNAs. The number of species with hits were tallied for each transcript (**Fig 1A**). Out of 4,709 sequences, 3,643 (77.4%) had no significant Blast hits and were denoted as species-specific. To exclude lncRNAs bearing spurious coding potential from downstream experiments, we trained and utilized a coding potential classifier using the annotated protein-coding transcripts [40]. Based on the model’s optimal cutoff, 3,636 transcripts were classified as non-coding (**Fig 1B**). Additional information regarding the localization features of lncRNAs was extracted through the analysis of their overlapping with annotated protein-coding elements of their genomic region (**Fig 1C and S3 Table)**.

### ZIKV infection modulates the expression of lncRNAs

**Fig 2:**
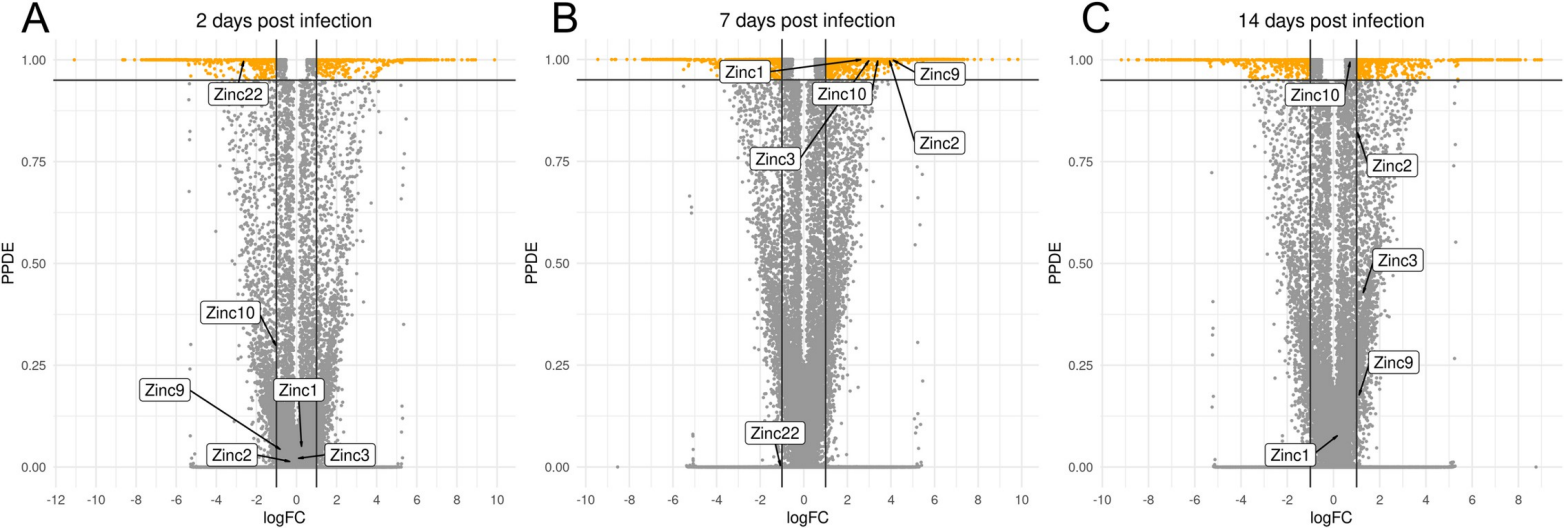
Volcano plot depicting differentially expressed transcripts in different time-points post Zika virus infection. **(A)** 2 days post-infection (dpi) with ZIKV, **(B)** at 7dpi, and **(C)** at 14dpi. The y-axis shows the posterior probability that each transcript was differentially expressed. Vertical lines indicate the applied log_2_FC threshold (|log_2_(FC)| ≥ 1), and the horizontal line indicates the statistical significance threshold, set at probability ≥95% (corresponding to a 5% false-discovery rate).

A publicly available RNA-Seq dataset [41] derived from whole-body *Ae. aegypti* specimens, comparing Zika virus infected to uninfected controls at 2, 7, and 14 days post-infection (dpi) [41], was analyzed to identify infection-responsive lncRNAs. Given our significant differential expression (DE) criteria of an absolute log_2_-fold change of at least 1 (equivalent to a two-fold change) and a minimum 95% posterior probability of belonging to the DE class, 823 (348 up- and 475 down-regulated upon infection), 1947 (1417 up- and 530 down-regulated) and 1303 (665 up- and 638 down-regulated) DE transcripts were found in the 2-, 7-, and 14-day comparisons, respectively (**Fig 2A–2C**). The lncRNA transcripts that displayed differential expression were termed Zika-modulated non-coding RNAs (Zinc). Five Zincs (*Zinc1*, *Zinc2*, *Zinc3*, *Zinc9*, and *Zinc10*) were selected for further functional studies due to their statistically significant upregulation, ranging between 6.6- to 17.1-fold, upon ZIKV infection at 7dpi (**Fig 2B**) and their species-specific nucleotide composition. In addition, the Zinc22 lncRNA was selected because of its significant downregulation at 2 dpi (**Fig 2A)** and its ovary-specific expression. Not wanting to limit our analysis to one category of lncRNAs with regard to their genomic localization, we chose three gene intron locating lncRNAs and three intergenic lncRNAs upstream/downstream of protein-coding genes, in sense/antisense orientation (**S1 Table)**.

**Fig 3:**
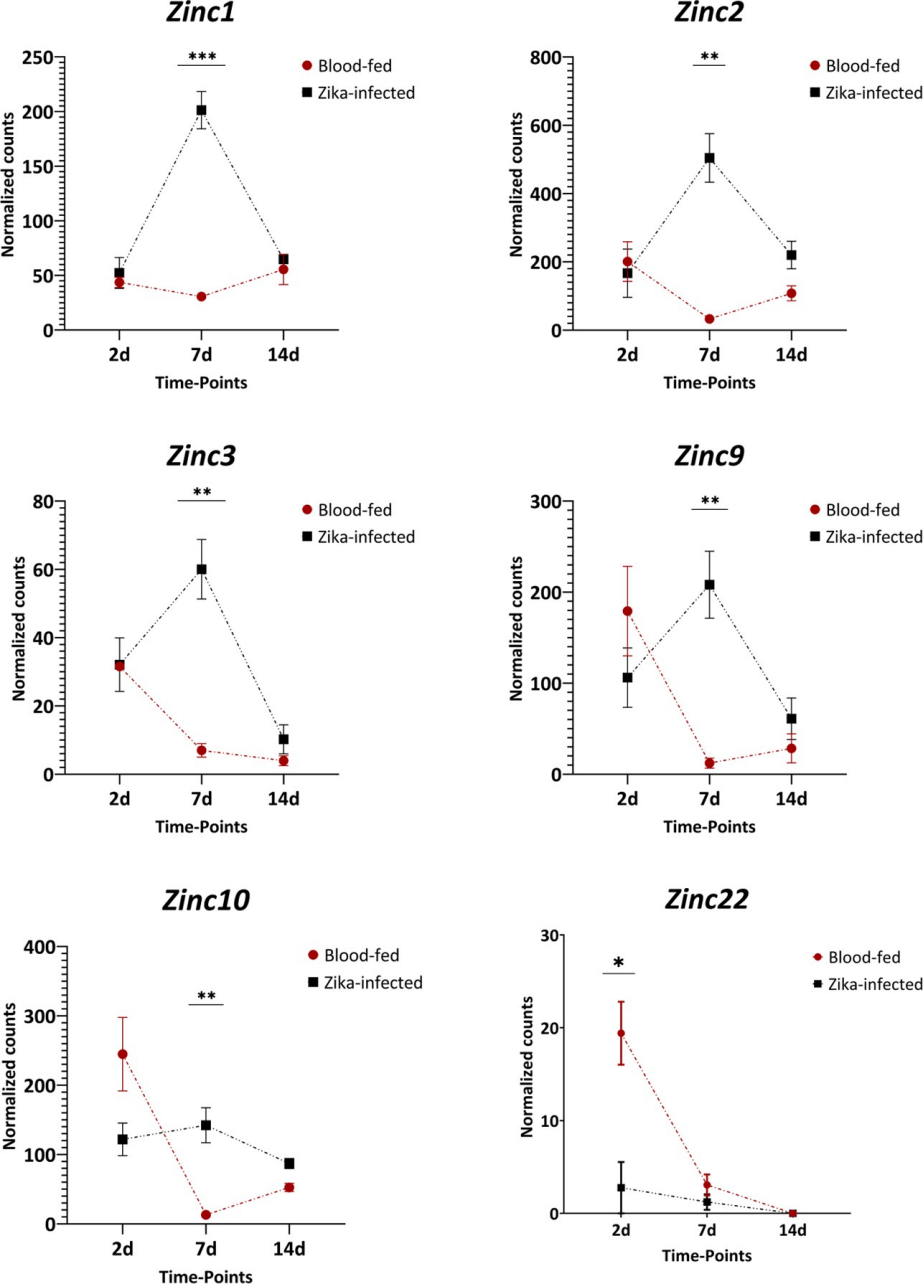
Expression of six Zinc lncRNAs in *Ae. aegypti* mosquitoes after a Zika virus-infected vs. non-infected blood meal. Data were collected at three different time points (2, 7, and 14 days post-blood-feeding). Comparisons were made between Zika virus-infected and non-infected samples at the same time point. On average, in infected samples, 7dpi *Zinc1* was upregulated 6.6-fold, *Zinc2* was up-regulated 15.4-fold, *Zinc3* 8.6-fold, *Zinc9* 17.1-fold, and *Zinc10* 10.9-fold, whereas *Zinc22* was down-regulated 7.0-fold at 2dpi. Each sample contained three biological replicates. Expression values are transcript-level read counts, normalized for library size using the median-by-ratio method. Bars indicate mean value ± SEM. The significance was determined by Student’s t-test. *:*P*<0.05, **:*P*<0.01, ***:*P*<0.001

In **Fig 2A–2C**, volcano plots present the state of differential abundance for each comparison; the six lncRNAs of interest are also annotated **(Fig 2A–2C and S1 Data**). According to the transcriptomic data that were analyzed, five of the six shortlisted DE lncRNAs were upregulated in infected mosquitoes at 7 dpi. Specifically, *Zinc1* displayed a 6.6-fold upregulation (*P*<0.001), *Zinc2* a 15.4-fold upregulation (*P*<0.01), *Zinc3* a 8.6-fold upregulation (*P*<0.01), *Zinc9* a 17.1-fold upregulation (*P*<0.01), and *Zinc10* an 10.9-fold upregulation (*P*<0.01). *Zinc22* showed a 7.0-fold downregulation in the infected mosquitoes at 2 dpi (*P*<0.05) (**Fig 3**). When we compared the expression levels of *Zinc22* in a detailed developmental dataset [42], this lncRNA emerged as ovary-specific and enriched in the insect’s post-vitellogenic stages. Validation of *Zinc22* expression by qPCR revealed a 10-fold upregulation in mock infected ovaries at 2 days post-blood meal (PBM), whereas it exhibited a 3-fold down-regulation upon ZIKV-infection (**S1 Fig**). The available transcriptomic data did not reveal tissue-specificity for the other *Zinc* genes.

### Silencing of Zinc lncRNAs influences mosquito permissiveness to virus infection

**Fig 4:**
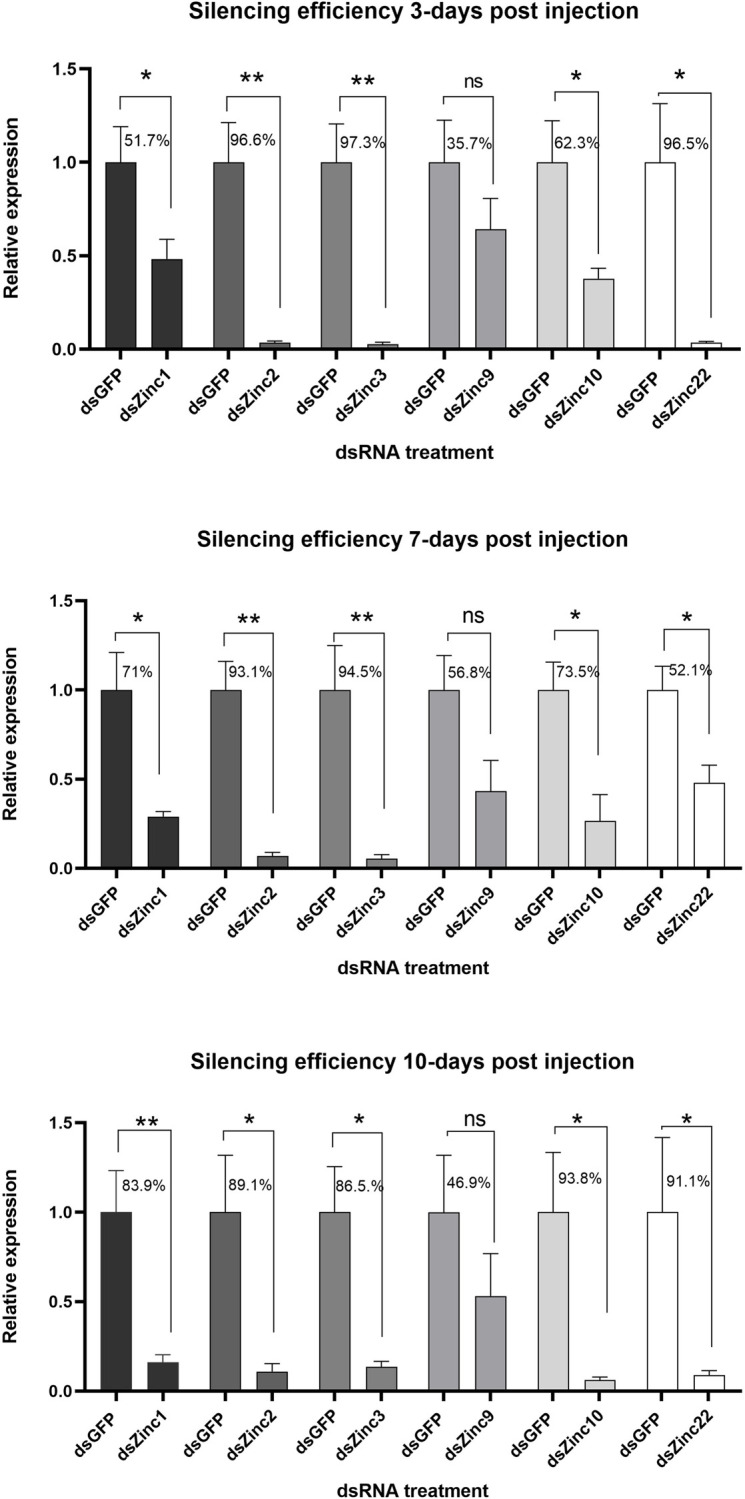
Zinc gene silencing efficiency. Relative quantification of *Zinc1*, *Zinc2*, *Zinc3*, *Zinc9*, *Zinc10* and *Zinc22* RNA abundance in Zinc-silenced vs GFP dsRNA-treated control mosquitoes. Whole mosquitoes were collected and pooled at three time-points (3-, 7- and 10-days) post-dsRNA injection. 3–5 biological replicates were performed for each Zinc and the average expression of GFP dsRNA treated replicates was set as 1 and the decreased dsZinc replicates, compared to GFP dsRNA, was measured. Samples were normalized using RNA abundance of the ribosomal RpS7 gene and are presented as the mean ± SEM. The significance was determined by Student’s t-test. *: *P*<0.05, **:*P<*0.01.

To elucidate the influence of Zinc lncRNAs on the mosquito’s susceptibility to virus infection, we used a standard RNAi approach based on dsRNA injection into the mosquito thorax. Target-specific dsRNA was generated against each of the Zincs and administered by microinjection into inseminated adult females at 7 days post-eclosion; control mosquitoes were injected with dsRNA against GFP (dsGFP). Silencing efficiency of Zincs was measured at three different time-points (3-, 7- and 10-days) post dsRNA injection. All Zincs showed silencing efficiencies from 51,7–83.9% for *Zinc1*, 89.1–96.6% for *Zinc2*, 86.5–97.3% for *Zinc3*, 35.7–56.8% for *Zinc9*, 62.3–93.8% for *Zinc10* and 52.1–96.5% for *Zinc22* (**Fig 4**). After confirming effective silencing, an infectious blood meal that contained a suspension of ZIKV mixed with human blood was provided to the injected mosquitoes at 2 days post-injection. Then, individual midguts and carcasses (i.e., the whole body, excluding the midgut) were dissected at 7 dpi and 10 dpi, respectively. The viral particles were extracted from the tissues, and the viral titer was measured through standard plaque assays in BHK-21 cells.

**Fig 5:**
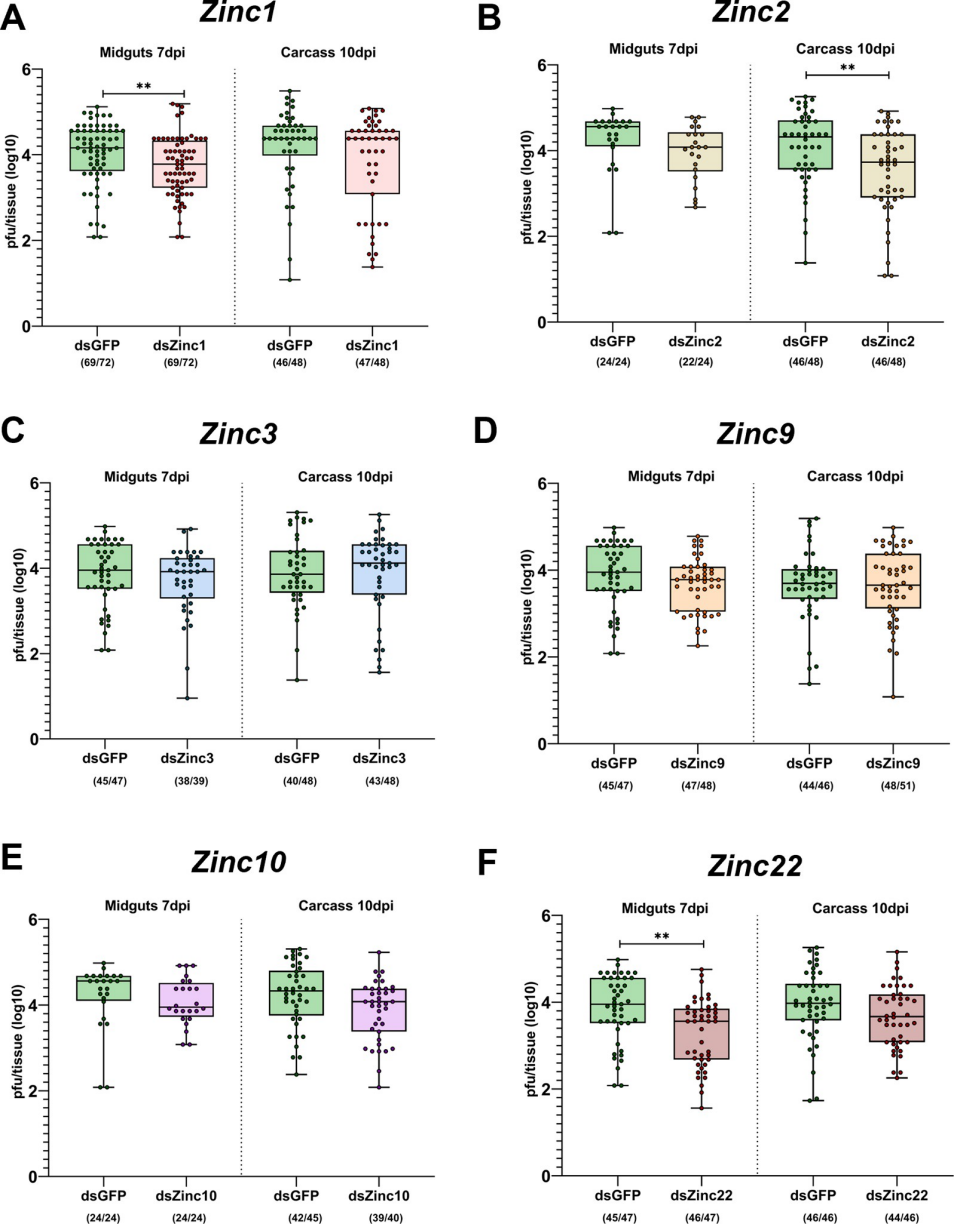
Effects of *Ae*. *aegypti* Zinc lncRNA silencing on ZIKV infection. Viral titers of Zika virus in midguts and carcasses obtained from dsRNA-treated mosquitoes at 7- or 10-days post-infection, respectively. Each panel corresponds to the comparison between Zinc-silenced mosquitoes and GFP dsRNA-treated controls: A) *Zinc1*-silenced, B) *Zinc2-*silenced, C) *Zinc3-*silenced, D) *Zinc9-*silenced, E) *Zinc10-*silenced, F) *Zinc22*-silenced. Each dot corresponds to the logarithmic number of plaque-forming units (pfu) that were counted per tissue and represents data from individual mosquitoes. The numbers in parentheses represent the number of virus-infected mosquitoes plotted against the number of mosquitoes checked. Data are presented as box plots in which the horizontal lines indicate the median value and whiskers show the minimum to maximum values. The significance was determined by Mann-Whitney U-test. **:*P*<0.01

Our results revealed that dsRNA-mediated silencing of *Zinc1*, *Zinc2*, and *Zinc22* resulted in a statistically significant decrease in the viral titers of Zika, whereas silencing of *Zinc3*, *Zinc9*, and *Zinc10* did not produce any significant changes in viral titer. Specifically, *Zinc1-*silenced midguts dissected at 7 dpi had a median titer of 10^3.7^ pfu, as compared to a median titer of 10^4.2^ (*P*<0.01) for the GFP dsRNA-injected control **(Fig 5A)**; *Zinc2* silenced mosquito carcasses dissected at 10 dpi had a median titer of 10^3.7^ pfu, as compared to 10^4.3^ (*P*<0.01) for the control **(Fig 5B)**; and *Zinc22*-silenced mosquito midguts dissected at 7 dpi had a median of 10^3.5^ pfu, as compared to 10^4^ (*P*<0.01) for the control (**Fig 5F**).

### Phenotypic impact of Zinc lncRNA silencing on fecundity and fertility

Given that the expression of the selected Zincs was modulated by infection with arboviruses, we next tested the hypothesis that their expression influenced reproduction in *Ae*. *aegypti*. Immunity and reproduction are two energy-demanding physiological processes that consume a significant proportion of an insect’s resources. To examine the possible involvement of selected Zincs in reproduction, we studied the impact of their RNAi-mediated silencing on three reproductive phenotypes: fecundity, fertility and progeny. dsRNA against either Zinc or GFP was administered by microinjection to inseminated adult female mosquitoes at 7 days post-eclosion, and 2 days later the injected mosquitoes were blood-fed on healthy mice. Their laid eggs were counted (fecundity assay) and the hatch rate was determined by comparing the percentage of successfully hatched larvae to the total number of eggs laid per mosquito (fertility assay). Progeny was defined as the number of live larvae produced by each mosquito.

**Fig 6:**
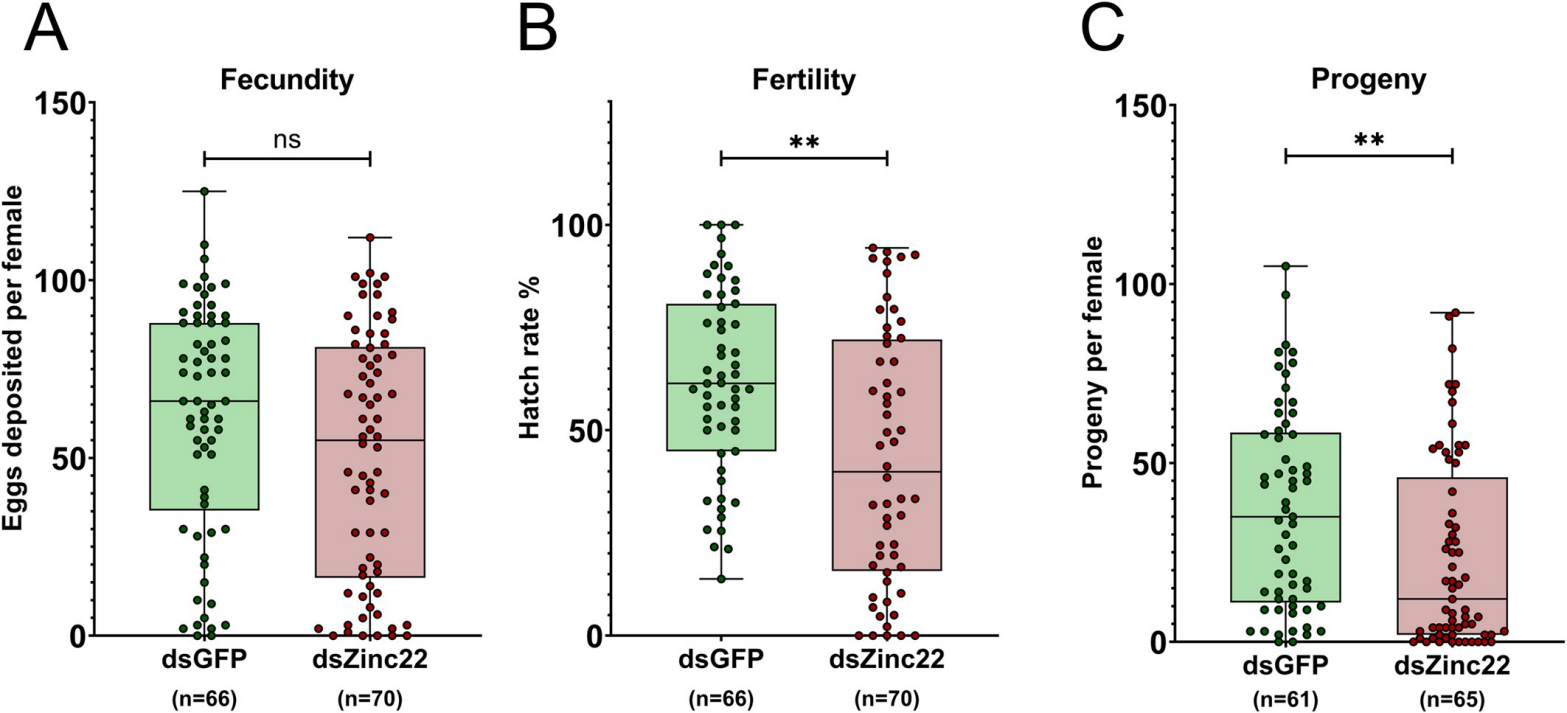
Impact of silencing of *Zinc22* lncRNAs on fecundity (oviposition), fertility (hatching) and progeny of *Ae*. *aegypti* mosquitoes. **(A)** Each dot corresponds to the number of eggs that were laid by each individual *Zinc22*-silenced or GFP dsRNA-treated female mosquito. **(B)** The fertility was defined as the percentage (%) of hatched larvae in relation to the total number of laid eggs per mosquito. **(C)** The number of offspring produced by each Zinc*-*silenced or GFP dsRNA-treated mosquito. Data are presented as box plots in which the horizontal lines indicate the median value and whiskers the minimum to maximum values. The significance was determined by Mann-Whitney U-test. **P<0.001.

We first examined the effect of *Zinc22*-silencing on reproductive phenotypes. The *Zinc22* is an ovary-enriched lncRNA that is upregulated by blood-feeding, but downregulated by Zika infection as shown by both RNA sequencing [41,42] and qPCR data (**S1 Fig)**. *Zinc22*-silenced mosquitoes showed a marginally non-significant decrease in the number of eggs laid when compared to the GFP dsRNA-treated control mosquitoes. Specifically, *Zinc22*-silenced females laid a median of 55 eggs, as compared to a median of 66 eggs for the GFP dsRNA-treated controls (*P* = 0.08) (**Fig 6A**). However, there was a significant decrease in the hatch rate of the *Zinc22* silenced females; showing a median egg hatch rate of 38.85%, whereas the GFP dsRNA-treated control mosquitoes produced eggs that had a median hatch rate of 61.40% (*P*<0.001) (**Fig 6B)**. Finally, *Zinc22*-silenced females produced a significantly lower number of offspring than did the GFP dsRNA-treated control females; *Zinc22*-silenced mosquitoes had a median of 12 offspring each, whereas each of the GFP dsRNA-treated control mosquito cohorts had a median of 35 offspring (*P*<0.001) (**Fig 6C**).

**Fig 7:**
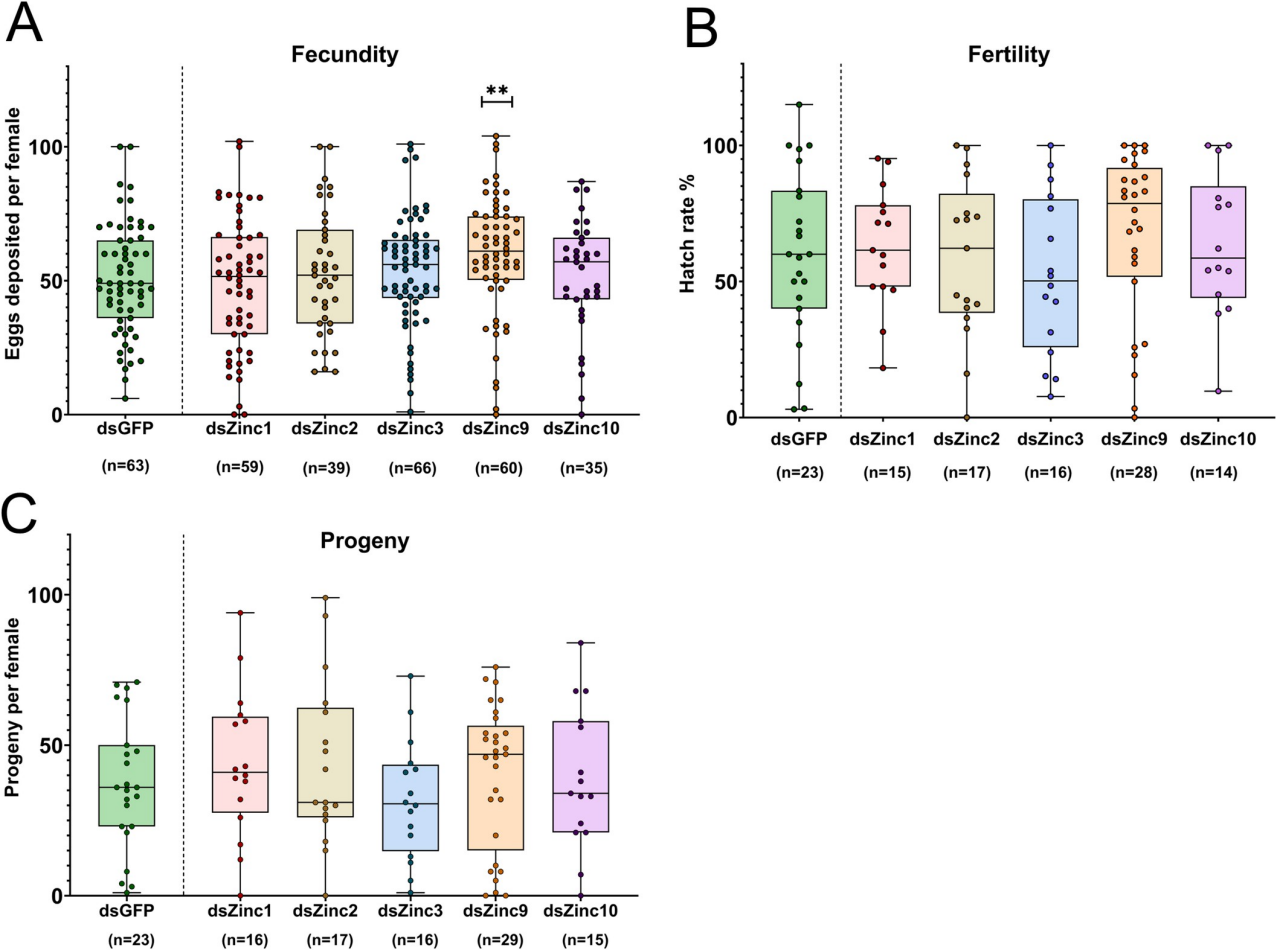
Impact of *Zinc1*, *Zinc2*, *Zinc3*, *Zinc9* and *Zinc10* lncRNA silencing on fecundity, fertility and progeny. **(A)** Each dot corresponds to the number of eggs that were laid by each individual Zinc-silenced or GFP dsRNA-treated female mosquito. **(B)** The fertility was defined as the percentage (%) of hatched larvae in relation to the total number of laid eggs per mosquito. **(C)** The number of offspring produced by each Zinc*-*silenced or GFP dsRNA-treated mosquito. Data are presented as box plots in which the horizontal lines indicate the median value and whiskers the minimum to maximum values. The significance was determined by Mann-Whitney U-test. **P<0.001.

Next, we expanded our silencing studies to another five Zinc lncRNAs. Silencing of *Zinc9* had a significant effect on fecundity (**Fig 7A),** while silencing of the other four Zincs had no effect, when compared to the GFP dsRNA-treated control mosquitoes, in either the fecundity, fertility or progeny assays (**Fig 7A–7C)**. Silencing of ds*Zinc9* resulted in an increased oviposition rate, to a median number of 61 laid eggs, as compared to a median of 49 eggs for GFP dsRNA-treated mosquitoes (*P*<0.001) (**Fig 7A**). An increase in the hatch rate of eggs was also evident for the *Zinc9*-silenced females, although this increase was not statistically significant. Specifically, eggs from the *Zinc9*-silenced females showed a median hatch rate of 79%, as compared to 60% for the GFP dsRNA-treated controls (**Fig 7B**). *Zinc9*-silenced females also produced more progeny compared to the GFP dsRNA-treated controls. Specifically, *Zinc9*-silenced mosquitoes had a median of 47 offspring each, whereas each of the GFP dsRNA-treated control mosquitoes had a median of 36 offspring which was not a statistically significant difference.

## Discussion

The role of lncRNAs in insect immunity and permissiveness to pathogens has been investigated in other species, particularly *D*. *melanogaster*, for which significant findings regarding its immune response against pathogenic viruses and bacteria have been reported [15,43,44]. Research has also been carried out in lepidopteran species such as *Bombyx mori* and *Pectinophora gossypiella*, in which specific lncRNAs have been implicated in the response to viral infection [45,46]. Despite the progress made in other species, lncRNA research in mosquito species has until now been limited and their implication in vector competence and immune responses to human pathogens has not been extensively addressed. Previous *Ae*. *aegypti* transcriptome studies have identified putative infection-regulated lncRNAs [41,47], and other studies have addressed specific *Ae*. *aegypti* lncRNAs that appear to interfere with arbovirus infection in cell lines [17,18,48]. Similarly, limited knowledge exists regarding the role of lncRNAs in *Ae. aegypti* reproduction. Transcriptomic analysis of *Ae*. *aegypti* ovaries has identified differentially expressed lncRNAs after a blood-meal [42,49], while studies in *Ae*. *albopictus* have revealed species-specific lncRNAs that influence fecundity and fertility [50].

Here we provide *in vivo* evidence regarding the influence of specific lncRNAs on the vector competence for Zika virus which to a significant degree is regulated by the innate immune system. We focused on vector competence and reproduction because of their significance for disease transmission and survival and their potential for the development of novel mosquito control strategies. Our screening revealed that the vast majority of *Ae*. *aegypti* lncRNAs (77.4%) displayed a high species specificity, as judged by their nucleotide composition. High species specificity is a common feature of insect lncRNAs, as has been observed in other related species such as *Ae*. *albopictus* [50], because of the absence of functional domains and a faster evolutionary rate of non-coding genes [51].

Subsequent analysis of ZIKV-infected *Ae*. *aegypti* samples revealed an abundance of lncRNAs that are differentially expressed at various time-points after infection. Silencing of *Zinc1*, *Zinc2* and *Zinc22* rendered the mosquitoes less permissive to virus infection while differences in viral titers were also observed upon silencing of other *Zinc* genes, although without statistical significance. The impact of *Zinc1*, *Zinc2* and *Zinc22* silencing indicates that these lncRNAs may act as, or regulate, pro-viral host factors that facilitate ZIKV infection. The effects of *Zinc1* and *Zinc22* silencing was mainly observed in the mosquito midguts at 7dpi suggesting that these lncRNAs may be involved in the early stages of ZIKV infection. The effect of *Zinc2-*silencing on infection of the carcass tissues at 10dpi suggests that its role in infection may be after the midgut stage of infection. Host factors that have been implicated in these processes are the Inosine-5′-monophosphate dehydrogenase (IMPDH) that promotes RNA synthesis of ZIKV and DENV [4] and TER94/VCP that is involved in uncoating of the ZIKV genome into the cytoplasm [52]. Another putative host factor that has been suggested to function as a DENV receptor is prohibitin [53]. Further studies are required to clarify the specific host factor mechanisms that *Zinc1*, *Zinc2* and *Zinc22* are implicated in.

The effects of *Zinc* silencing may also indicate their potential roles as negative regulators of the mosquito anti-viral defense. The Toll and JAK/STAT immune signaling pathways, that have been shown to respond to ZIKV infection [4], comprise negative regulators such as Cactus Toll [54] and PIAS, respectively [9]. While no ncRNAs have been linked to the regulation of either pathway in *Ae*. *aegypti*, the *D*. *melanogaster* lncRNA *CR11538* has been shown to suppress the Toll pathway [43], and miRNAs such as *miR958*, *miR964* and *miR317* are also a negatively regulating Toll signaling [55–57]. While these findings do not imply that the same interactions occur in *Ae*. *aegypti*, they do introduce a novel research target for investigation.

Given that vector competence and immunity have been connected to reproduction [24,25,27,28,39], as evidenced by the reproduction-immunity trade-off, a tempting question was whether the ZIKV-regulated *Zinc* lncRNAs had any impact on *Ae*. *aegypti* reproductive functions. Interestingly, *Zinc22* silencing resulted in a significant reduction in egg hatching, as well as a decrease in the number of progeny. Considering that *Zinc22* expression is downregulated upon ZIKV infection at 2 days PBM, while its silencing results in a suppression of infection in midguts at 7dpi it is likely that it may be involved in reproduction-immunity trade-offs. Based on the silencing phenotypes, one can hypothesize that a decrease in Zinc22 expression results in a stronger immune response against the virus and the trade-off of this energetically costly process is a reduction in reproduction. The trade-offs between the two systems have been shown to be regulated by several endocrine signals, and one of them is the production of 20-hydroxyecdysone (20E) which is essential for the production of yolk protein precursors (YPPs) involved in vitellogenesis and egg development. The 20E is produced in the fat body upon hydroxylation of ecdysone, a hormone that is produced by the female ovarian follicles in response to a blood-meal (reviewed in [32]. Upregulation of ecdysone increases mosquito reproductive ability, while its decrease its immune competence [58]. *Zinc22* was identified as an ovary-specific lncRNA and may therefore be involved in the regulation of metabolic processes of ovaries. The apparent roles of *Zinc22* as a positive regulator of reproduction and a negative regulator of immunity suggests it could be implicated in such processes.

It is noteworthy that silencing of *Zinc9* produced an increase in mosquito fecundity, and to a lesser extent, fertility. Although *Zinc9* was selected for our studies as a potential immune-related lncRNA, its silencing did not have any significant effect on infection with ZIKV. This could be a result of reduced silencing efficiency (35.7–56.8%), as compared to the silencing effects of dsRNA treatments for other Zincs. Additional experiments evaluating the influence of *Zinc9* on the resistance to other pathogens, including other arboviruses and bacteria, should be conducted to further investigate its potential role in immunity. Considering its demonstrated effects on reproduction, any immune-related outcome could suggest its involvement in trade-offs.

In conclusion, our studies point at the potential influence of four mosquito lncRNAs on reproduction and vector competence. As regulators of these critical processes, these lncRNAs have the potential for development of novel vector and disease transmission control strategies. For example, reproductive enhancement through transgenic overexpression of a reproduction-regulating lncRNA could provide a fitness advantage to genetically modified pathogen-resistant mosquitoes [59,60]. Similarly, lncRNAs that either boost or disable antiviral immunity could be exploited to create transgenic virus-resistant mosquitoes. Refractory mosquitoes can be generated either by overexpressing antiviral effector genes or by inactivating pro-viral host factors [61,62].

## Materials and methods

### Ethics statement

This study was conducted in accordance with the recommendations in the Guide for the Care and Use of Laboratory Animals of the National Institutes of Health, the Animal Care and Use Committee (ACUC) of Johns Hopkins University, and the institutional Ethics Committee. The Institutional Animal Care and Use Committee (IACUC) approved the protocol RA21H388. Mice were used for the mosquito rearing and reproductive assays. Anonymous, commercial blood from human donors was used for virus infection assays in mosquitoes.

### lncRNA annotation

Non-coding RNA (ncRNA) sequences (XR accessions) were obtained from the *Ae*. *aegypti* GCF_002204515.2 gene models. Blastn [63] was executed locally against RefSeq RNAs of Hexapoda (TaxID 6960) for each transcript at default settings. Hits on opposite strands (+/-) as well as within *Ae*. *aegypti* (TaxID 7159) were filtered out.

FEELnc [40] was used to perform species-specific estimation of the coding potential of all non-coding RNAs. In brief, FEELnc_filter.pl was used to filter out ncRNAs overlapping with the exons of protein-coding transcripts (XM accessions) in the sense orientation as well as ncRNAs smaller than 200 nucleotides. The parameters for FEELnc_codpot.pl were established to build a coding potential estimation model using protein-coding transcripts (mode = shuffle). Using 10-fold cross validation, 0.432 was identified as the model’s optimal cutoff point to denote coding and non-coding transcripts (i.e., at this point the model’s sensitivity and specificity is maximized). The coding potential of the filtered ncRNAs (n = 3929) was estimated, classifying 3,636 (92.5%) of them as non-coding. In addition, FEELnc_classifier.pl was used to annotate ncRNA genes regarding their genomic localization relative to overlapping/closest protein-coding genes. Overlapping instances were annotated as sense/antisense, nested/overlapping and intronic/exonic, and intergenic ones were annotated as sense/antisense, same-strand/convergent/divergent and upstream/downstream. ncRNA genes exhibiting no neighboring genes within 100kb were annotated as distal (n = 416).

The R packages ggplot2 [64] and networkD3 [65] were used to render the plots shown in **Fig 1**. The annotation of all *Ae*. *aegypti* ncRNAs regarding their species-specificity, coding-potential, and genomic localization with respect to protein-coding transcripts is provided in **S3 Table**.

### Transcriptome analysis

The RNA-Seq dataset produced and described by Etebari *et al*. [41] was retrieved from the Sequence Read Archive. FASTQC [66] and Cutadapt [67] were used for quality control and removal of adapters and low-quality bases, respectively. The *Ae*. *aegypti* GCF_002204515.2 AaegL.5.0 genome assembly and respective gene models were used to create indices for the STAR v2.7.9a aligner [68] (a splice-aware index, taking into account the dataset’s read length) and for the RSEM v1.3.1 quantification tool [69]. Genomic alignment of paired-end reads was conducted with STAR and parameterized according to the ENCODE options stated in its manual, and subsequently RSEM quantification was conducted. EBSeq v1.30.0 [70], which is tailored to isoform-level analyses, was used to identify DE transcripts. In brief, transcript-level raw counts were used to obtain library normalization factors with the default median-by-ratio method [71]; they were then subjected to empirical Bayes testing for 15 iterations (*maxround* argument), ensuring that the model’s hyper-parameter estimations converged. Differential expression was called at a 5% target false-discovery rate, setting *FDRMethod* argument at “hard” and disabling the *Threshold*_*FCRatio* filter. Transcripts exhibiting a two-fold expression change and at least a 95% posterior probability of being DE (PPDE) were considered to be DE.

Median-by-ratio normalized read counts were utilized to render plots of differentially expressed transcripts were produced using the ggplot2 R package. **S1 Data** provides the entire DE analysis results.

### Mosquito rearing

*Aedes aegypti* mosquitoes (Liverpool strain) were maintained on a 10% sucrose solution in the insectary of the Johns Hopkins Malaria Research Institute at 27°C under 85% relative humidity and a 12h light/12h dark cycle.

### Cell cultures

An *Ae*. *albopictus* C6/36 cell line was used for the ZIKV (Cambodia, FSS13025) propagation. Cells were grown in minimal essential medium (MEM, Gibco, Carlsbad, CA, USA) with 10% heat inactivated FBS, 1% L-glutamine, 1% penicillin-streptomycin, and 1% non-essential amino acids at 32°C with 5% CO_2_. A cell line of baby hamster kidney BHK-21 cells was used for the plaque assays. They were maintained on Dulbecco’s modified Eagle’s medium (DMEM, Gibco, Carlsbad, CA) supplemented with 10% FBS, 1% L-glutamine, 1% penicillin-streptomycin, and 5 μg/ml plasmocin (Invitrogen, Carlsbad, CA) at 37°C with 5% CO_2_.

### dsRNA synthesis and gene silencing

Total RNA was extracted from ten 7-day old *Ae*. *aegypti* females using Trizol (Invitrogen, Carlsbad, CA). cDNA was synthesized from total RNA using an oligo-dT primer and random hexamers with the MMLV reverse transcriptase kit (Invitrogen, Carlsbad, CA). Total cDNA was used to amplify Zinc lncRNAs by PCR, using gene-specific primer sets with the T7 RNA polymerase promoter (TAATACGACTCACTATAGGG) attached to their 5’ ends. GFP gene was similarly amplified using a GFP plasmid as a template (**S2 Table**). Amplified PCR products were purified with a Zymo DNA cleanup kit (Zymo Research, Irvine, CA). A total of 1 μg of PCR product was used to synthesize dsRNA using a NEB T7 HiScribe kit (NEB, Ipswich, MA) according to the manufacturer’s instructions. dsRNA was purified using isopropanol-sodium acetate precipitation. dsRNA was dissolved in sterile distilled water, and the concentration was measured with a Nanodrop spectrophotometer (ThermoFischer Scientific, Waltham, MA). The final concentration of the dsRNA was adjusted to 3 μg/μL.

One-week-old inseminated female mosquitoes were anesthetized on a cold block and intrathoracically injected with 69 nL of 3 μg/μL dsRNA against each of the Zinc genes or GFP as a control. Mosquitoes were reared for 2 days in the insectary to recover the injection before any downstream experiment. For the purpose of analyzing silencing efficiency whole mosquitoes were collected at three time-points (3-,7-,10-days) post-dsRNA injection and 5 females were pooled for each biological replicate. 3–5 biological replicates and two technical replicates per lncRNA were performed. PCR conditions are described in the Real-Time qPCR section.

### Zika infections and plaque assays

C6/36 cells in T25 flasks were infected with ZIKV at a multiplicity of infection (MOI) of 0.5. After 4–5 days of incubation at 32°C with 5% CO_2_, infected cell culture medium was harvested and mixed with human red blood cells (RBC) supplemented with human serum containing 10 mM ATP (50% RBC, 40% ZIKV medium, 10% serum, 1% ATP). Injected females at 2 days post-injection were starved overnight and transferred to small (16 oz) paper cups. They were allowed to feed for 30 min on a blood-virus mixture at 37°C on a single glass artificial feeder. Fully engorged females were recovered by sorting and maintained on 10% sterile sucrose solution. Midguts from individual females at 7 dpi and carcasses from individual females at 10 dpi were dissected on ice and kept at −80°C. Each experiment had at least two biological replicates, and each replicate included at least 18 individual females.

Mosquito midguts and carcasses were homogenized in 300 μl DMEM with a Bullet Blender (Next Advance, Inc., Averill Park, NY) with glass beads. They were then centrifuged at 10,000 × *g* for 3 min, and 50 μl of the filtered supernatants was used for plaque assays with BHK 21 cells in 24-well plates. After the plates were incubated for 4 days for ZIKV, the plaques were visualized by staining with 1% crystal violet and counted under a microscope. Differences in viral intensity and prevalence of infection in samples dissected from Zinc-silenced and GFP dsRNA-treated females were compared by using a nonparametric Mann-Whitney U test and Fisher’s exact test, respectively.

### Reproductive assays

#### Oviposition

Four days after blood-feeding, individual female mosquitoes were placed in 50-mL Falcon tubes that contained a moist filter paper at the bottom. A range of 35 to 70 mosquitoes were included in each *Zinc-silenced*/GFP dsRNA treated sample. The mosquitoes were left in the Falcon tubes in the insectary for 2 days to allow egg laying to occur. Moisture was regularly added to the tubes to keep the filter paper wet. Then mosquitoes were removed from the Falcon tubes, and the total number of eggs deposited by each individual was photographed and counted using ImageJ software [72]. Mosquitoes that deceased during the oviposition process were excluded from the analysis.

#### Hatching assay

The filter papers with eggs were collected, and the eggs were dried and then stored in sealed Petri dishes that contained a wet cotton ball as a source of humidity. The eggs laid by 14 to 56 individual mosquitoes were included in each *Zinc-*silenced/GFP dsRNA-treated sample. They were stored in separate Petri dishes and three days (72h) after egg-laying, 30ml of hatching broth was added to each dish. Hatching broth consisted of a fish food pellet diluted in 1L of autoclaved water. The eggs were then incubated in the hatching broth for up to 14 days. Hatching broth was replaced with fresh broth every 4 days. Larvae that hatched from each egg batch were counted daily and removed from the Petri dishes. The hatch rate was estimated as the percentage of emerged larvae divided by the total number of eggs laid:

$$hatch\;rate\;=\frac{\;emerged\;larvae}{\;laid\;eggs}\times 100\%$$

### Real-time qPCR

Ovaries were dissected from mosquitoes that were either: (i) non-blood fed (NBF), (ii) blood-fed with a blood mixture from healthy humans (50% RBC, 40% MEM, 10% serum, 1% ATP) at 48h PBM or (iii) blood-fed with human blood infected with Zika virus (50% RBC, 40% Zika medium, 10% serum, 1% ATP) at 48h PBM. The dissected ovaries were pooled into three groups of six ovaries for each condition and stored in 100μL of TRIzol reagent (Invitrogen, Carlsbad, CA). Total RNA was extracted according to the TRIzol manufacturer’s instructions. The integrity of the RNA was assessed by agarose gel electrophoresis. Total RNA was treated with DNase I (ThermoFisher Scientific, Waltham, MA), and 1μg of RNA was reverse-transcribed to cDNA by using oligo-dT primers and MMLV-RT (Invitrogen, Carlsbad, CA).

Gene-specific primers (**S2 Table**) were used for qPCR amplification. Amplification and analysis were carried out using the StepOnePlus real-time PCR system (Applied Biosystems, Warrington, UK). The final reaction volume was 15μl when using the ABI SYBR Green supermix. The PCR program was as follows: hold at 95°C for 10 min, 95°C for 15 s, and 60°C for 1 min, repeated for 40 cycles. The specificity of the SYBR Green PCR signal was further confirmed by melting curve analysis. The relative abundance of the gene transcripts was normalized and calculated by comparison to the ribosomal protein S7 gene (AAEL009496) as an endogenous reference using the Livak (2^-ΔΔCt^) method.

## Supporting information

S1 Fig Expression of *Zinc22* in ovaries of mosquitoes that were fed with sugar (NBF) or a blood meal mixed with uninfected (mock) or ZIKV-infected cell culture medium (Zika). Expression data were generated by Real-Time qPCR. Values were normalized with the ribosomal gene Rps7. Each sample included three biological replicates. Bars indicate mean value ± SEM. The significance was determined by Student’s t-test. *:*P*<0.05
(TIF)

S1 Data Differential expression analysis of three different time-points in mosquitoes fed with infected (Zika) vs non-infected blood meal. Data include coding and non-coding transcripts that were either up- or down- regulated according to RNA seq data.
(XLSX)

S1 Table Genomic features and accession numbers (ID) of Zinc lncRNAs. (XLSX)

S2 Table Primers used in the present study. (XLSX)

S3 Table Annotation of all *Ae*. *aegypti* ncRNAs regarding their species-specificity, coding-potential, and genomic localization with respect to protein-coding transcripts. (XLSX)
