# Supplementary figures and images for: Long non-coding RNAs regulate Aedes aegypti vector competence for Zika virus and reproduction
Source: PLoS Pathog. 2023 Jun 15;19(6):e1011440. doi: 10.1371/journal.ppat.1011440 (PMC10306205; doi:10.1371/journal.ppat.1011440)

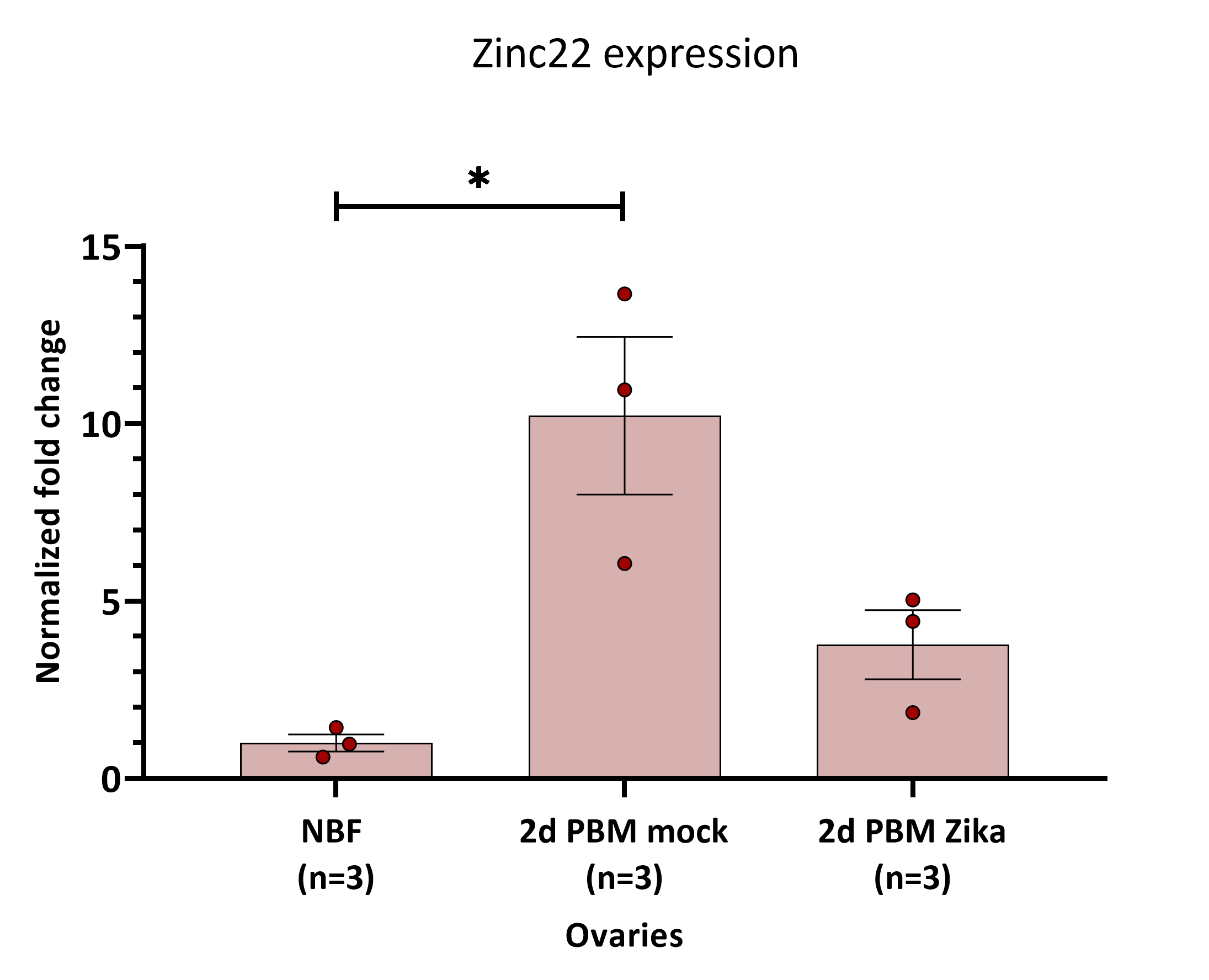

Supplement: S1 Fig — Expression data were generated by Real-Time qPCR. Values were normalized with the ribosomal gene Rps7. Each sample included three biological replicates. Bars indicate mean value ± SEM. The significance was determined by Student’s t-test. *:P<0.05 (TIF) [file ppat.1011440.s001.tif]
